# Taxonomic discoveries bridging the gap between our knowledge and biodiversity

**DOI:** 10.3897/phytokeys.94.23887

**Published:** 2018-01-29

**Authors:** Xiao-Hua Jin, Yun-Hong Tan, Rui-Chang Quan

**Affiliations:** 1 Southeast Asia Biodiversity Research Institute, Chinese Academy of Sciences, Yezin Nay Pyi Taw 05282, Myanmar; 2 Institute of Botany, Chinese Academy of Sciences, Beijing, 100093, China; 3 Centre for Integrative Conservation, Xishuangbanna, Tropical Botanical Garden, Chinese Academy of Sciences, Mengla Yunnan 666303, China

Southeast Asia includes four overlapping biodiversity hotspots: Indo-Burma, Philippines, Sundaland and Wallacea (Myers et al. 2000; Sodihi et al. 2004). Southeast Asia covers about 4.5 million km^2^, which is approximately 3 % of earth’s total land area. There is, however, approximately 20 to 25 % of Earth’s higher plant species in this area (Myers et al. 2000). It is crucial to understand the biodiversity for conservation and sustainable development in the shadow of climate change and growth of economics and population.

Biological surveys and scientific research of biodiversity have a long history in Southeast Asia and several hypotheses for biogeography have been proposed (e.g. Che et al. 2010; Hou and Li 2017). However, the species richness of biodiversity is far underestimated. Taxonomy, including discoveries of new taxa, taxonomic revision and inventory, is the precondition of our conservation and sustainable development. Although frontiers of taxonomy and systematics biology, integrated taxonomy and genomics are main trends, the taxonomic work of checklist, flora and description of new taxa are far from sufficient in Southeast Asia. Many species will become extinct before we know that they even exist in Southeast Asia. Although it is a daunting task, it is extremely urgent to investigate, understand and conserve our biota.

In order to understand and conserve the biodiversity in Southeast Asia, the Southeast Asia Biodiversity Research Institute (SEABRI) was established by the Chinese Academy of Sciences in 2014. It is an international scientific research and education organisation managed by the Xishuangbanna Tropical Botanical Garden (XTBG). With financial and personnel support from Chinese Academy of Sciences, SEABRI seeks to substantially improve our understanding and conservation of biodiversity in Southeast Asia by cooperation with all CAS institutes, international agencies and government of ASEAN countries.

This special issue of Phytokeys, entitled “Plant diversity in Southeast Asia” represents a new effort by SEABRI to promote awareness of the biodiversity and its conservation in the region. We are here firstly focusing on taxonomic discoveries to bridge the gap between our knowledge and diversity. Twelve articles in this issue mostly involve the description of new species from botanical surveys in the region. They include two new species of *Oreocharis* (Gesneriaceae) and a new species of *Didymocarpus* (Gesneriaceae) from Vietnam, a new species of *Aristolochia* (Aristolochiaceae), a new species of *Dendrobium* (Orchidaceae), a new species of *Gastrodia* (Orchidaceae), a new species of *Hedychium* (Zingiberaceae) and two new species of *Trivalvaria* (Annonaceae) from Northern Myanmar, a new species of *Primulina* (Gesneriaceae) from southwest China and seven species of *Begonia* (Begoniaceae) from Northern Vietnam and Southern China. The description of the little known species, *Begonia kingdon-wardii* (Begoniaceae) in Myanmar was also included. Results of molecular phylogenetics of tribe Neottieae (Orchidaceae) are also reported. Most studies are financially supported by the CAS (2015CASEABRI005, Y4ZK111B01).
